# Physical Properties of Biological Entities: An Introduction to the Ontology of Physics for Biology

**DOI:** 10.1371/journal.pone.0028708

**Published:** 2011-12-27

**Authors:** Daniel L. Cook, Fred L. Bookstein, John H. Gennari

**Affiliations:** 1 Department of Physiology and Biophysics, University of Washington, Seattle, Washington, United States of America; 2 Department of Biological Structure, University of Washington, Seattle, Washington, United States of America; 3 Division of Biomedical and Health Informatics, University of Washington, Seattle, Washington, United States of America; 4 Department of Statistics, University of Washington, Seattle, Washington, United States of America; 5 Faculty of Life Sciences, University of Vienna, Vienna, Austria; Max Planck Institute for Evolutionary Anthropology, Germany

## Abstract

As biomedical investigators strive to integrate data and analyses across spatiotemporal scales and biomedical domains, they have recognized the benefits of formalizing languages and terminologies via computational ontologies. Although ontologies for biological entities—molecules, cells, organs—are well-established, there are no principled ontologies of *physical properties*—energies, volumes, flow rates—of those entities. In this paper, we introduce the Ontology of Physics for Biology (OPB), a reference ontology of classical physics designed for annotating biophysical content of growing repositories of biomedical datasets and analytical models. The OPB's semantic framework, traceable to James Clerk Maxwell, encompasses modern theories of system dynamics and thermodynamics, and is implemented as a computational ontology that references available upper ontologies. In this paper we focus on the OPB classes that are designed for annotating physical properties encoded in biomedical datasets and computational models, and we discuss how the OPB framework will facilitate biomedical knowledge integration.

## Introduction

The biotechnology enterprise, from laboratory bench to bedside, depends on the interpretation of the meaning of data at all structural levels from molecules to whole organisms. Furthermore, emerging methods of multiscale biosimulation increasingly integrate this knowledge across biophysical domains; e.g. connecting fluid kinetic knowledge with chemical kinetic knowledge (see Table 1). For example, building on pioneering mathematical modeling methods (e.g., Hodgkin and Huxley [1], Guyton [2]), international research efforts such as the IUPS Physiome [3], the EU Virtual Physiological Human [4], “systems biology” [5], and “executable biology” [6] aim to share data and integrate models across all time scales, spatial scales, and biophysical domains. Such integrative computational efforts are recognizing the value of biomedical ontologies for annotating the biophysical content of their underlying mathematical biosimulation code [7]. Unfortunately, much biomedical data, and many models, remain “siloed” in the purview of specific biomedical disciplines and laboratories where, even if made available, are hidden from other investigators.

**Table 1:** Processes in different biophysical domains.

*Biophysical domain*	*Biophysical process*
fluid kinetics	blood flow, respiratory gas flow
solid kinetics	musculoskeletal mechanics, myocardial contraction
chemical kinetics	cell metabolism, gene expression, cell signaling
electrochemistry	transmembrane ion flow, nerve action potential
diffusion kinetics	alveolar gas exchange, cellular calcium dynamics
heat kinetics	metabolic heat production, body surface cooling

Biophysical processes occur in different biophysical domains and over time spans and spatial scales.

### Need for a reference ontology of biophysics

Central to sharing data and knowledge in support of such integrative efforts are biomedical ontologies [8], [9] that formalize and standardize the terms concepts, and relationships used in biomedical research and practice. On-line ontology repositories such as the Open Biological and Biomedical Ontology (OBO) Foundry [10] and the National Center for Biomedical Ontology (NCBO ) BioPortal [11] are clearing houses for ontologies that encompass human anatomy (e.g., Foundational Model of Anatomy (FMA [12]), animal anatomy (e.g., mouse anatomy [13]), cells and cellular anatomy (e.g., Cellular Component Ontology, as part of the Gene Ontology [14]), macromolecules (e.g., UniProt [15]), and small chemicals (e.g., ChEBI [16]). Other ontologies classify clinical concepts (e.g., SNOMED-CT [17], openGALEN [18]), investigational methods (e.g., Ontology of Biomedical Investigation [19]), physiochemical concepts (e.g., IUPAC Gold Book [20]), and biological phenotypes [21] (e.g., Phenotypic Quality Ontology (PATO [22], Mammalian Phenotype Ontology [23]).

At base, what we know about the physical world, and biomedical processes, is tied to measures of physically observable states and state properties that become the biomedical data, the variables of analytical models, and the subjects of written and verbal discourse. Whereas some ontologies (e.g., PATO, OBI, IUPAC Gold Book [20], Systems Biology Ontology [24]) include classes for biophysical attributes (e.g., pressure, expression rate, electrical potential) these classes are defined and classified only in a piecemeal, informal, and domain-specific manner that fails to include a “…deep understanding [of] how numbers and the physical world work…” [25]. Thus, our goal is to develop a reference ontology of biophysical properties and biological processes that will be useful to: (1) annotate the biophysical content of biomedical datasets, (2) annotate and implement analytical models of biomedical processes [7], [26]–[28], and (3) semantically resolve and map the biophysical content of biomedical ontologies.

### Background: Maxwell on encoding physical meaning

The measurement, analysis, and simulation of biological processes depend on observable physical and thermodynamical quantities such as force, charge, and energy. Available physical ontologies [29], [30] targeting engineering systems strive, understandably, to encompass engineering models in terms of their mathematics—“the natural language” of physics. However, for the biomedical domain, we sought a declarative semantics based on the *physical meaning* of quantities on the premise that it is more critical to know that a model variable or experimental datum is a *fluid pressure* or *tensile stress* rather than that it is a *scalar* or a *tensor*. To represent physical meaning, we have adopted a classificatory approach proposed by the physicist James Clerk-Maxwell (1831–1879) in a short note to the London Mathematical Society, “On the Mathematical Classification of Physical Quantities” [31].

Maxwell observed that “in recent times that we have become acquainted with so large a number of physical quantities that a classification of them is desirable”, and proposed:

- “One very obvious classification of quantities is founded on that of the *sciences* in which they occur…[such as]…action of heat on bodies…magnetic induction…electro-static induction”.
- “…the classification which I now refer to is founded on the *mathematical or formal analogy* of the different quantities and not on the matter to which they belong.”
- “…and the [third is] a mathematical classification of quantities.”

The novel aspect of our approach, and the focus of this paper, is the classification of physical quantities according to their physical meaning as established by their formal analogies across biomedical sciences. For example, Oliver Heaviside (1850–1925) proposed the “hydraulic analogy” (see Wikipedia: “Hydraulic analogy”) in which fluid flow in a pipe is analogous to electrical current flow in a wire; fluid pressure to electrical voltage; and fluid flow resistance to electrical resistance. Extensions and formalizations of such analogies pervade and organize the science of system dynamics as formulated for engineering systems [29], [32] and for biological networks [33]–[35]. Our motivation is that the annotation and reuse of biological data and analytical models depends first on establishing the physical meaning of observable quantities based on ontological relations that determine, secondarily, their mathematical relations; e.g., a distance traveled is the temporal integral of the traveler's speed.

We are motivated by utilitarian goals of facilitating and expediting the annotation and cross-referencing of physics-based analytical models and data in the realm of biomedicine and strive to represent those concepts that are the basis for quantitative analysis of biophysical entities and processes. Thus, OPB it is not intended to represent physical “reality” as advocated for some biomedical ontologies [36] rather we intend to represent the concepts and laws that have long served as the basis for quantitative explanations of how the biological world works (see [25].

### Scope and goals

As an ontology of the abstract concepts of classical physics, systems dynamics, and thermodynamics, the OPB's top class is OPB:*Physical analytical entity* which we define as “…a formal abstraction of the real world created within the science of classical physics for describing and analyzing physical entities, attributes, and processes.” We aim to encompass biological entities and processes (Table 1) that are usefully represented in terms of the transformation and flow of thermodynamic energy. Thus, the OPB is based on concepts that hold at the spatial and temporal scales of biophysical processes, and that are described in textbooks of classical physics [37], [38], biology [39], [40], biomechanics [41], [42], and chemical biophysics [43], [44]. OPB does not encompass evolutionary, social, or psychological processes (for which thermodynamic energy is undefined) nor does it encompass quantum or relativistic physics (which are rarely invoked for biomedical processes). Furthermore, OPB is not intended to fully recapitulate the axiomatic basis of physics as a theoretical framework.

Whereas the foundational theory of the OPB encompasses both discrete systems analysis using ordinary differential equations (ODE) and continuum systems analysis using partial differential equations (PDE), the first version of the OPB is targeted solely to discrete systems analysis. Thus, it deals strictly with the physical properties of discretized entities whose values are spatial integrals over spatial elements, and physical dependencies that can be written as ordinary differential equations.

Our long term goals for the OPB are ambitious. In this paper, as a starting point, we focus exclusively on those OPB classes that establish the physical meaning of observable quantities, based on the framework laid out by Maxwell.

## Materials and Methods

Each OPB class bears a machine-readable, unique identifier (e.g., OPB_00528) in addition to a human-interpretable class name (e.g., OPB:*Physical property*). This to avoid confusion where commonly used names of physical properties often refer to the physical entity it quantifies (e.g., a spatial region “volume” has a scalar measure “volume”), or where terms are used in more than one physical domain. Thus, we provide unambiguous class names and human-readable definitions in the “comment” relations of the ontology.

As we recognize the value of upper ontologies (UO) for alignment and interoperability, we strive to define OPB classes in a manner consistent with UOs such as Basic Formal Ontology (BFO) [45], General Formal Ontology-Biology (GFO-Bio) [46], and Descriptive Ontology for Linguistic and Cognitive Engineering (DOLCE) [47]. However, we defer to future work the formal alignment of OPB classes to UO classes, except to informally note where such class-class correspondences may occur. We have built the OPB class taxonomy using the Web Ontology Language (OWL) [48] and the Protégé 4.1 ontology editor.

Our aim in this paper is to offer a high-level view of how we have defined classes to capture the physical meaning of physical properties; thus, not all subclasses will be exposed, nor will we discuss other top-level OPB classes (OPB:*Physical entity*, OPB:*Physical process entity*, OPB:*Physical dependency*) that are currently under development. The first version of OPB (v. 1.0) comprises only those classes required for annotating biophysical data and model variables, as are outlined in this paper. It is available for download from the NCBO BioPortal repository site [49].

## Results

As a roadmap to the remainder of this paper, we begin by providing an overview of the three main classes in the OPB that provide the context for physical properties per Maxwell's framework. Next, we extend this organization by using a systems dynamics framework that encompasses important dependency relationships among physical quantities that occur across multiple biophysical domains. We then provide details and examples of the main OPB classes as drawn from a wide variety of physical domains and scales.

**Figure 1:**
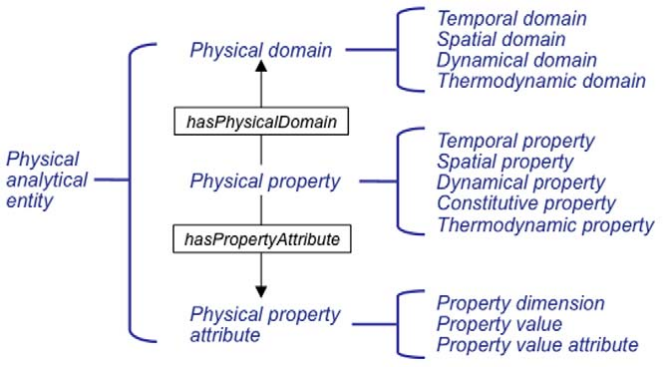
OPB main classes. The top-most OPB class is OPB:*Physical analytical entity* (at the right) which has, following a suggestion by Maxwell [31], subclasses OPB:*Physical domain*, OPB:*Physical property*, and OPB:*Physical property attribute* (center) with subclasses of each shown at the left. Each OPB:*Physical property* class is assigned to one or more OPB:*Physical domain* classes (by a *hasPhysicalDomain* relation; gray arrow) and to one or more OPB:*Physical property attribute* classes (by *hasPropertyAttribute* relations).

As shown in Figure 1, the three main subclasses of OPB:*Physical analytical entity* that we use to define the semantics of observable quantities are:

- OPB:*Physical domain* classes that correspond to Maxwell's “sciences”,
- OPB:*Physical property attributes* that correspond to property dimensions, forms, etc.,
- OPB:*Physical properties* that correspond to Maxwell's “physical quantities”.

### Physical domains and property attributes

OPB:*Physical domain* classes are useful for classifying OPB:*Physical property* according to the physical science to which the class applies using the OPB:*hasPhysicalDomain* relation. For example OPB:*Fluid pressure has_domain* OPB:*Fluid kinetic domain* as would OPB:*Fluid volume*. It is envisioned, therefore, that domain-specific sub-ontologies can be easily created by excluding classes that apply to other domains, and that annotations made against OPB classes can be checked to affirm domain consistency (e.g., to exclude annotations of a portion of fluid with a physical property of the OPB:*Solid kinetic domain* such as OPB:*Solid stress*).

OPB:*Physical property attributes* classes are for annotating the particular mathematical form of instances of data or model variables in a particular application. For example, property instances can be distinguished according to their: 1) mathematical form (e.g., scalars, vectors, differentials), 2) physical dimensions (e.g., length, time, charge), and 3) numerical values as scaled to particular units of measure (e.g., meter, second, coulomb). For example, attributes of a OPB:*Solid stress* value are a OPB:*Property value mathematical form* attribute (scalar, vector, or tensor) and a OPB:*Property value coordinate basis* attribute (e.g., OPB:*Spherical coordinate system*) with which the value is defined. Such attributes have been the focus of other ontologies including EngMath [30] and Ontology on Property [50].

We have implemented OPB:*Property dimension* subclasses of OPB:*Physical dimension* to represent physical dimensions (e.g., angle, length) that are the basis of dimensional analysis [51] and of systems of units of measure (e.g., radian, centimeter) [52]. We have implemented OPB:*Physical dimension* subclasses as the set of base dimensions (length, time, mass, charge, temperature, luminosity, angle) as proposed by Schadow (as “kind of quantity”) [52] from which a coherent set of derived property dimensions for other physical properties may be derived as products of base dimensions raised to integer (both positive and negative) powers (e.g., velocity = length•time^−1^, pressure = force•length^−2^, volume = length^3^).

### A system dynamical framework based on physical meaning

**Figure 2:**
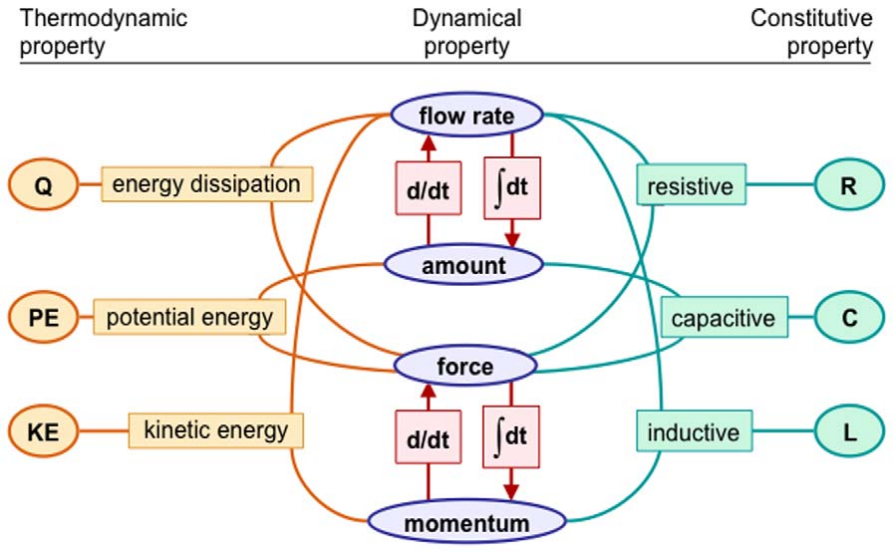
Foundational physical theory of OPB. Framework in which physical properties (ovals) are linked by quantitative dependency relations (rectangles) between the quantitative magnitudes of properties. For example, Ohm's law is a resistive dependency between electrical current (I, a flow rate), voltage differential (V, a force), and electrical resistance (R, a resistive constitutive property). This schema applies, wholly or in part, to properties in various physical domains (e.g., fluids, electricity, chemistry) and are the basis for analogies between property types. (Q = rate of heat dissipation, PE = potential energy, KE = kinetic energy, R = resistance, C = capacitance, L = inductance.).

Recognizing cross-domain analogies as developed in the field of system dynamics, the OPB is built on a conceptual framework (Figure 2) that identifies three classes of physical property: thermodynamic property (OPB:*Thermodynamic property*), dynamical property (OPB:*Dynamical property*), and constitutive property (OPB:*Constitutive property*). Within a given physical domain, each property is defined by its quantitative dependency relationships (OPB:*Physical dependency*) with others properties. For example, a resistance property (R) characterizes a resistive dependency (OPB:*Resistive dependency*) between a flow rate and a force whereas a thermodynamic dependency defines energy dissipation (Q; a kind of OPB:*Energy flow rate*) in terms of the same pair of flow rate and force properties.

**Figure 3:**
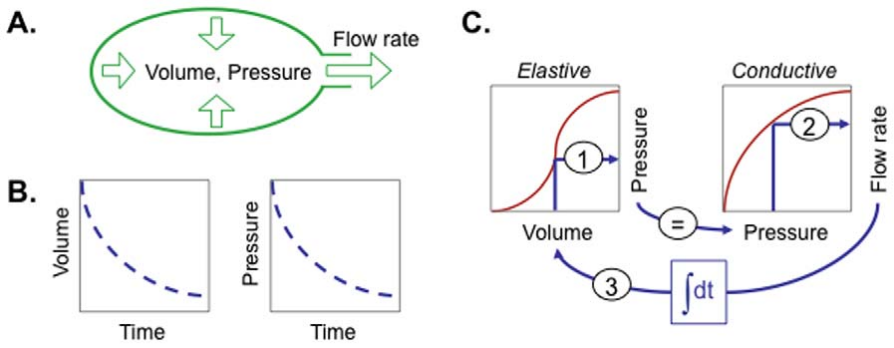
Simple example of a dynamical model. (A) Two-element “Windkessel” model for fluid flowing from an elastic vessel such as a balloon, lung, or blood vessel. (B) Positive pressure in the vessel due to tension in the vessel wall drives fluid from the vessel which decreases both the volume and pressure as a function of time. (C) An iterative algorithm (blue arrows) can simulate the time course of changing volume, pressure, and flow rate in terms of a temporal integral dependency, an elastive dependency (the reciprocal of a capacitive dependency), and a conductive dependency (the reciprocal of a resistive dependency).

This schema applies, for example, to “Windkessel” models (“air chamber”, in German; e.g., [53], [54]) of fluid flow into and out of elastic vessels such as balloons, lungs, or blood vessels (Figure 3A). Thus, if one considers the fluid contained in the Windkessel to be a discrete, homogenous entity, one is concerned with three discrete dynamical properties—*volume*, *pressure*, and *volume flow rate*—such that positive fluid pressure expels fluid from the vessel, and the reduced volume reduces pressure as the vessel wall relaxes (Figure 3B). The time course of the deflation can be computationally simulated using a simple algorithm (Figure 3C; blue arrows) in terms of temporal integral and constitutive dependencies: 1) the *volume* at any time determines *pressure* according the *elastive dependency* that is a reciprocal of a *capacitive dependency*, 2) the *pressure* determines a *flow rate* according to the reciprocal of a constitutive *resistive dependency*, and 3) a *temporal integral* of the *flow rate* determines how much the *volume* changes per unit time as indicated by the box labeled “∫dt”.

Physics and system dynamics are concerned with the measurement, analysis, and simulation of physical processes. The OPB maps the concepts of classical physics into a declarative and computable form for the annotation of biophysical datasets and models. Thus, we have sought to define and classify an ontology class for each physical property and for physical dependency relations that are relevant to the annotation and analysis of multidomain biophysical systems. We first expand the OPB:*Dynamical property* class with subclasses for different dynamical domains. We then introduce thermodynamical property classes and dependencies, and lastly discuss constitutive properties of constitutive dependencies.

### OPB Physical property classes

We define OPB:*Physical property* as “…an attribute of a physical entity, property, or process that has a quantitative value that could be measured by a physical device, or computed from such measures.” It follows then that an instance of a physical entity (e.g., your heart) can have instances of more than one kind of physical property (your heart can have a location and a volume), yet an instance of a physical property can be an attribute of only a single instance of physical entity (the location of your heart is an attribute only of your heart, not of mine). Examples of OPB:*Physical property* classes include OPB:*Spatial location* (e.g., of a heart) and OPB:*Chemical amount* (e.g., of glucose in a cell). OPB:*Physical property* extends UO classes such as BFO:*Quality* and GFO:*Quality*.

OPB distinguishes the property itself from its value at a moment in time. For example, the portion of blood in a vessel has *volume* (OPB:*Fluid volume*) which has a *value* (OPB:*Property value*) that is measureable in a specified unit of measure at a particular time. Although one can argue that a portion of blood having zero volume no longer exists as a portion of blood, the OPB supports the implementation convention of databases and analytical models that entities and their properties persist despite having property values that may imply their nonexistence.

Here we consider three important OPB:*Physical property* subclasses: OPB:*Dynamical property*, OPB:*Constitutive property*, and OPB:*Thermodynamic property* each of which is defined in terms of the others by quantitative dependencies (OPB:*Physical property dependency*) that represent the laws and definitions of classical physics. In this first version of OPB, we rely on informal concepts of dependencies for purposes of defining and distinguishing physical properties while deferring to a later OPB version the formal implementation of dependency classes (OPB:*Physical property dependencies*) and their formal relations to properties until later versions of OPB.

#### Dynamical properties

Dynamical properties are defined as “…a property of an energy-bearing physical entity whose value determines the amount or rate of change of the amount of thermodynamic energy inhering in the entity”. Values of dynamical properties depend on the values of other dynamical properties (Figure 2) according to a network of dependencies that are the basis of the systems dynamics approach to physical analysis.

**Figure 4:**
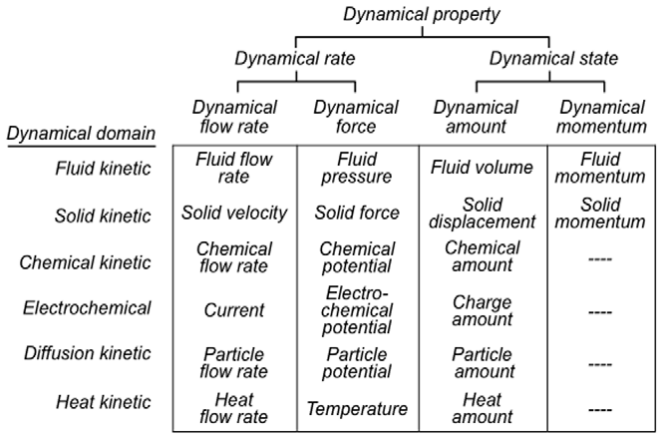
OPB:*Dynamical property* subclasses. Each subclass is cross-product of a one of four OPB:*Dynamical property* classes with one of the six OPB:*Dynamical domain* subclasses (except for OPB:*Dynamical momentum* subclass).

The OPB:*Dynamical property* subclass hierarchy (top, Figure 4) distinguishes rate properties (OPB:*Dynamical rate property*) that are the rates of change of conjugate state properties (OPB:*Dynamical state property*) of the same instance of a discrete dynamical entity according to the schema in Figure 2. Figure 4 also displays the OPB:*Dynamical property* subclasses as they apply to physical entities of each of the currently supported dynamical domains.

Examples of amount/flow rate temporal integrals are:

- In a Windkessel model of blood flow, the volume (OPB:*Fluid volume*) of a portion of blood is the temporal integral of the net volume flow rate (OPB:*Fluid volume flow rate*) of blood entering and leaving the portion of blood.
- The amount of a portion of a chemical (OPB:*Chemical amount*) is the temporal integral of the net flow rate (OPB:*Chemical flow rate*) due to all sources and sinks for the chemical including formation/destruction in reactions and influx/efflux transport across the boundary of the portion of chemical (e.g., transmembrane glucose transport into and out of cytoplasm).

Examples of momentum/force temporal integrals are:

- The momentum (OPB:*Pressure momentum*) of a portion of blood is the temporal integral of the net pressure (OPB:*Fluid pressure*) applied to the boundary of a portion of blood. Pressure imbalances thus accelerate the blood flow rate and, thus, give it momentum.
- The momentum (OPB:*Solid momentum*) of a solid entity is the temporal integral of the net of forces (OPB:*Solid force*) operating on the solid entity. Force imbalances accelerate the solid entity according to Newton's law (acceleration = force/mass) and impart momentum.

In addition to amounts and momenta being the temporal integrals of flow rates and forces, respectively, amounts and momenta are subject to conservation constraints that are not shown in Figure 2. Thus, the OPB includes dependency classes for *OPB:Conservation of momentum* and OPB:*Conservation of amount* whose subclasses apply to mass, charge, and space.

#### Thermodynamic properties

Feynman [38] reminds us that, although we may not know exactly what energy is, physicists define different kinds of thermodynamic energy in terms of dynamical state and rate properties (as in Figure 2). For example, the potential energy of a linear spring is defined as one-half of the product of the springs axial force (OPB:*Solid force*) times its axial displacement (OPB:*Solid displacement*). If the spring's compressive force accelerates a mass, the spring's potential energy (OPB:*Potential energy amount*) is converted into kinetic energy of the moving mass (OPB:*Kinetic energy amount*). Just as for dynamical properties, there are energy subclasses corresponding (as appropriate) to amounts of energy for each of the dynamical domains (e.g., OPB:*Fluid kinetic energy*). Biological examples of thermodynamic properties include:

- OPB:*Fluid kinetic domain*: A portion of blood has fluid potential energy due to its pressure (including gravitational “head”), and has kinetic energy according to how fast it is flowing. The pressure potential energy is converted into kinetic energy as the pressure accelerates fluid flow rates through a vessel. Viscous shear forces within the blood flow dissipates the blood's total energy into heat.
- OPB:*Chemical kinetic domain:* A portion of chemical (as dissolved in a cell's cytoplasm) has an amount of chemical potential energy (but no kinetic energy) proportional to the chemical potential energy of each molecule times the number of molecules. The amount of chemical potential energy of such a portion of molecules can be converted into energy of portions of other species during biochemical reactions of metabolic and cell signaling pathways. Variously defined chemical potential energies are fundamental to the analysis of chemical reaction kinetics particularly in complex chemical networks [44].

The mathematical definitions of energy properties in terms of dynamical properties are represented as OPB:*Thermodynamic definition* classes such as OPB:*Energy definition* classes such as, for example, one definition of total energy (OPB:*Total energy amount*) of a moving object that is the sum of its kinetic energy (OPB:*Kinetic energy amount*) and its potential energy (OPB:*Potential energy amount*) with respect to potential fields within which it exists as well as the energy attributed to its internal composition. This constrains these components of total energy to be conserved such that total energy can only be changed by the influx or efflux of energy (OPB:*Energy flow rate*) to or from other entities. Systems dynamics and network thermodynamics are sciences concerned with the exchange and transformation of potential and kinetic energy within and between energetic entities as constrained by the (conservation of energy). Examples:

- OPB:*Fluid kinetic domain*: A pressurized portion of blood converts its potential energy (OPB:*Fluid potential energy amount*) to kinetic energy (OPB:*Fluid kinetic energy amount*) when the pressure accelerates the blood through a conduit.
- OPB:*Solid kinetic domain*: The elastic potential energy (OPB:*Strain potential energy amount*) to kinetic energy (OPB:*Solid kinetic energy amount*) when a stretched muscle accelerates a bone.
- OPB:*Chemical kinetic domain:* The chemical potential energy (OPB: *Chemical potential energy amount*) of a portion of one chemical (e.g., a metabolic substrate) is converted to the chemical potential energy of a product chemical (e.g., ATP).

Definitional and conservational dependencies for dynamical and thermodynamic properties provide certain constraints on their values, yet how such property values change in time during a physical process is determined by constitutive dependencies that are based, ultimately on empirical observations of constitutive properties such as mass density, resistivity, and permittivity.

#### Constitutive properties

Constitutive properties (of constitutive dependencies) characterize empirically derived physical laws that depend on the structural composition (e.g., distributions of mass, charge, etc.) and material properties of participants in a process. From elementary physics, the electrical circuit laws for ideal resistors, capacitors, and inductors generalize to constitutive laws that apply to energy flows in other physical domains. Thus we have three subclasses of OPB:*Constitutive path dependency* (“path” because each dependency describes a path by which energy is exchanged or dissipated):

- OPB:*Resistive dependency* generalizes the dependence of electrical potential (E, an OPB:*Dynamical force*) across an electrical conductor on the electrical current (I, an OPB:*Dynamical flow rate*) flowing through the conductor of resistance, R, so that, according to Ohm's law, E = IR.
- OPB:*Capacitive dependency* generalizes the dependence of the amount of electrical charge (Q; an OPB:*Dynamical amount*) stored by an electrical capacitor on the potential difference (E; an OPB:*Dynamical force*) and the capacitance, C, of the capacitor. Thus, according to an electrical analog of a linear Hooke's law, Q = EC,
- OPB:*Inductive dependency* generalizes the dependence of the potential difference (E; an OPB:*Dynamical force*) across an inductor of inductance, L, and the time-rate of change of the current, I (an OPB:*Dynamical flow rate*), passing through the conductor with inductance, L, so that E = LdI/dt.

**Figure 5:**
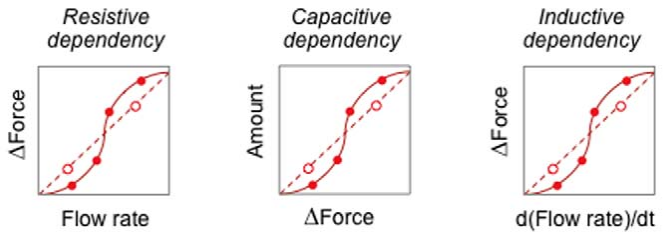
Examples of linear and non-linear dependencies. OPB:C*onstitutive physical dependency* subclasses are quantitative relationships between pairs of dynamical properties. One or more parameters are required for mathematical functions being used to compute the shape of the dependencies.

Just as for OPB:*Dynamical property* classes, OPB:*Constitutive path dependency* classes have subclasses for each physical domain (although inductance dependencies apply only to solid, fluid, and electrical domains). Each of the three constitutive path dependencies are empirically determined dependencies between the dynamical properties that are “players” in the dependency—a resistive dependency is a relation between a flow rate and a force differential, for example. Because electrical circuit theory generally assumes “ideal” circuit elements, linear, proportional (Figure 5, dotted lines) parameters are sufficient: resistance (R; OPB:*Resistance property*), capacitance (C; OPB:*Capacitance property*), and inductance (L; OPB:*Inductance property*), respectively. Furthermore, biophysical analyses (as in Figure 3C) commonly employ inverted dependencies (OPB:*Conductance dependency*, OPB:*Elastance dependency*) whose proportionality properties (G; OPB:*Conductance property* and E; OPB:*Elastance property*, respectively) are reciprocals of resistance and capacitance. Although such proportional approximations to constitutive dependencies are sufficient for some analytical purposes, in general the nonlinearity of biological constitutive dependencies require more complex algebraic functions of multiple parameters to be fitted to empirical data (the “data points” in Figure 5).

Constitutive path dependencies apply to the dissipation or transformation of energy of only a single kind, or within a single physical entity. However, biological processes are largely a story of control, transformation and exchange of different energy kinds between different physical entities; for example the transformation of chemical potential energy into the mechanical energy of muscle contraction. Such energy coupling is critical for multiscale, multidomain biological processes which occurs via three kinds of OPB:*Constitutive coupling dependencies* according to constitutive coupling properties (OPB:*Constitutive coupling proportionality*):

- OPB:*Transducer dependency*: The chemical potential energy of high-energy phosphate compounds is converted into elastic potential energy of myofibrillar proteins. Transducer dependencies describe the thermodynamically balanced conversion of energy of one kind into a different kind. A microphone transduces sound energy into electrical energy according to a parameter called the transducer modulus (OPB:*Transducer ratio*).
- OPB:*Transformer dependency:* The forearm acts as a lever by which the force of muscle contraction is transformed into a force that lifts a handheld weight. Transformer dependencies describe the thermodynamically balanced transfer of energy of a single kind from one physical entity to another. An (ideal) electrical transformer transforms the voltage and current of one electrical coil into that of another according to a transformer ratio (OPB:*Transformer ratio*).
- OPB:*Transactor dependency*: The level of neurotransmitter in a synaptic cleft controls the contractile force of a biceps muscle. This is action at a distance where one property controls the value of another with no (or minimal) regard to thermodynamics. For example, the location of an accelerator pedal controls the acceleration of an automobile. The proportionality constant of a transactor has type OPB:*Transactor proportionality*

## Discussion

The OPB is by no means complete as we recognize that ontologies must constantly grow and evolve to satisfy real-world use-cases. The current version of OPB, and this paper, focus on establishing the physical meaning of physical quantities as OPB:*Physical property* classes that we continue to test against use-case applications. As we encounter new use-cases, we may need to evolve, the OPB class tree by extending or adding new classes.

### Foundational theory

Just as mereotopology, the topological science of part-whole relations [55], is a foundational theory of structural ontologies (e.g., the FMA), system dynamics and thermodynamics [29], [32], [34], [56] are foundational theories of the OPB. The result, for the OPB, is a self-referential semantic system (as in Figure 2) in which the meaning of a class lies in its multiple, simultaneous dependencies on other classes. Because such physical dependencies hold simultaneously throughout the occurrence of a biophysical process, no OPB:*Dynamical property* class can be declared as an ontological “primitive” with respect to others. For example, Newton's Second Law takes on three forms (i.e., f = ma, m = f/a, and a = f/m) that define each quantity in terms of the other two. The implication of this, in practice, is that biophysicists are free to observe some properties and infer (by calculation) the values of other (unobservable) properties from dependency relations as in Figure 2.

### Use-case applications

Our long term goal is to use the OPB as a resource for ontology-based biological modeling [7], [26], [28] and for annotating data resources across scales and domains. Toward this, we have used the OPB classes for merging multiscale heart-rate control models [28], merging cardiovascular dynamics models across computational platforms [57], [58], and for more general model reuse tasks implemented by our SemGen application [59], [60]. Furthermore, we have used the OPB for the annotation and intermapping of the biophysical content of biosimulation models in the realm of cardiovascular dynamics and metabolic systems [28], [57], [58], [60], [61]–[62], and have demonstrated how OPB temporal and dynamical property classes can be used to annotate observable attributes of biological processes [63]. The OPB may also serve as a reference ontology for mapping biophysical content across existing biomedical ontologies such as PATO [22], SBO [63], and OBI [19], as well as for the biosimulation models available in the CellML model repository, BioModels repository [64], and NSR-Physiome repository [65].

### Future directions

Here we have described the OPB's approach to representing classes of physical property that are of concern to biomedical research guided by use-cases that require the annotation of biomedical datasets and biosimulation models. Based on these results and the system dynamical framework we have established, we will continue to represent the physical entities that are the bearers of physical properties. As our approach is based on thermodynamic and classical physics, we will classify biological objects—hearts, molecules, cytoplasm, etc.—as OPB:*Dynamical entities* (subclasses of OBP:*Physical entity*) that are defined as “…the bearer of portions of thermodynamic energy whose amounts are determined by the values of the dynamical physical properties of the dynamical entity.” Following that, we formally implement OPB:*Physical dependency* classes in terms of role-playing physical properties with the ultimate goals of axiomatizing dependency relations to support automated reasoning and for providing computational “pseudocode” for implementing dependencies in simulation models. These implementations will then be the basis for formalizing a thermodynamic theory of biological processes (as classes of OPB:*Physical process*) that encompasses theories of mereotopology, system dynamics, and thermodynamics. This theory will include key principles of the Process Ontology [66] and will be designed for formal reasoning over complex biological processes.

### Summary

We have here outlined the major class structure of the Ontology of Physics for Biology that represents key physical concepts of systems dynamics and thermodynamics as they occur in biomedical sciences. The OPB is a computational ontology intended for annotating the biophysical content of biomedical knowledge resources including databases, analytical models, and other biomedical ontologies. As a reference ontology, the OPB is orthogonal and complementary to, existing biomedical ontologies as it defines physical concepts according to the principles of classical physics. We have developed a declarative representation of the formal structure of system dynamical theory in terms of observable physical properties and the physical laws by which the values of those properties depend upon one another. Thus OPB:*Physical property* classes are based on analogical relations first suggested by Maxwell and are mapped to the dynamical domains of concern to biomedicine. To test the utility of OPB, we have used it as a reference ontology to annotate and semantically analyze a broad range of multiscale/multidomain data and modeling resources.
